# Enhancing Post-Surgical Rehabilitation Outcomes in Patients with Chronic Ankle Instability: Impact of Subtalar Joint Axis Balance Exercises Following Arthroscopic Modified Broström Operation

**DOI:** 10.3390/medicina60020328

**Published:** 2024-02-15

**Authors:** Ji-Myeong Park, Sang-Ho Han, Byeong-Chae Cho, Se-Min Lee, Mal-Soon Shin, Jae-Ho Yu, Ho-Jin Kim, Hyun-Dong Noh, Min-Suk Cho, Myung-Ki Kim

**Affiliations:** 1 Sports Medical Research Center, Daechan Hospital, 590 Inju-daero, Namdong-gu, Incheon 21570, Republic of Korea; come90@korea.ac.kr (J.-M.P.); doctortrust@naver.com (S.-H.H.); hyundi0813@naver.com (H.-D.N.); 2 Nowon Samsung Orthopedics, 456 Nohae-ro, Nowon-gu, Seoul 01762, Republic of Korea; bc.jo81@gmail.com (B.-C.C.); sem8282@gmail.com (S.-M.L.); 3 Department of Global Sport Studies, Korea University, 2511 Sejong-ro, Jochiwon-eup, Sejong 30019, Republic of Korea; malsoon@korea.ac.kr; 4 Department of Physical Therapy, Sunmoon University, 70, Seonmun-ro 221beon-gil, Tangjeong-myeon, Asan-si 31460, Republic of Korea; naresa@sunmoon.ac.kr; 5 Department of Sports and Exercise Medicine, Biomedical Science, Korea University, 2511 Sejong-ro, Jochiwon-eup, Sejong 30019, Republic of Korea; ho101jin@hanmail.net (H.-J.K.); cmspassion@naver.com (M.-S.C.)

**Keywords:** anterior talofibular ligament, arthroscopic modified Broström operation, chronic ankle instability, subtalar joint axis balance exercises

## Abstract

*Background and Objectives:* This study aimed to evaluate the effects of subtalar joint axis-based balance exercises on the anterior talofibular ligament (ATFL) thickness, ankle strength, and ankle stability after an arthroscopic modified Broström operation (AMBO) for chronic ankle instability (CAI). *Materials and Methods:* The study included 47 patients diagnosed with CAI who underwent AMBO and were randomly divided into three groups: control (*n* = 11), general balance exercise (*n* = 17), and subtalar joint axis balance exercise (*n* = 19), regardless of the affected area. Participants in the exercise rehabilitation group performed exercises for 60 min twice a week for six weeks, starting six weeks after AMBO. ATFL thickness, ankle strength, and ankle dynamic stability were measured using musculoskeletal ultrasonography, Biodex, and Y-balance test, respectively, before and after treatment. *Results:* Compared with the remaining groups, the subtalar joint axis balance exercise group had reduced ATFL thickness (*p* = 0.000), improved ankle strength for eversion (*p* = 0.000) and inversion (*p* = 0.000), and enhanced ankle stability (*p* = 0.000). *Conclusions:* The study results suggest that subtalar joint axis-based balance exercises may contribute to the early recovery of the ankle joint after AMBO.

## 1. Introduction

Ankle joint injuries are caused by walking on uneven ground, sudden changes in direction, sudden stops, and incorrect landings after jumping in daily life and sports activities [1]. Ankle injuries are among the most prevalent sports injuries, accounting for 10–30% of all sports injuries [2]. The most frequent injuries include lateral strain injuries, which occur through excessive suppression movements of plantar flexion, inversion, and rearfoot of the ankle, and external rotation movements of the leg [3]. Subtalar joint instability is a common complication of ankle sprains [4,5], and excessive inversion of the ankle and internal rotation of the talus result in lateral ankle sprains [5,6].

Among ankle injuries, inversion sprain accounts for over 85% of cases, whereas injuries of the anterior talofibular ligament (ATFL) comprise 70–80% [1]. According to the National Health Insurance Statistical Yearbook in the Republic of Korea in 2022, a total of 2,043,134 patients visited the hospital for an ankle sprain [7]. Ankle sprains are classified into grades I, II, and III based on severity. Grade I injuries involve mild strain and ligament relaxation with local swelling, tenderness, and mild loss of function. Grade II injuries entail partial ligament tear with swelling, pain, tenderness, and partial loss of function. Grade III injuries are severe complete ligament ruptures, characterized by severe pain, swelling, and instability [8]. Approximately 10–50% of patients with ankle sprains demonstrate chronic ankle joint sprain symptoms, including chronic pain or repetitive reinjury [9,10]. Moreover, functional and mechanical instability for more than six months [11] and frequent ankle sprains cause physical deformation of the subtalar joint axis, ligaments, and the hindfoot [5]. However, secondary chronic ankle instability (CAI) with recurrent ankle sprains can persist in 10–30% of severe cases with ligament tears [12]. Patients with CAI have a high prevalence of cartilage degradation, anterior bone impingement, and soft tissue impingement [13]. The lack of interaction between the talus and calcaneus can lead to abnormal movement of the subtalar joint, causing a decline in foot and lower limb function and resulting in abnormal deformation of the foot structure. Ultimately, this condition increases the risk of ankle sprain or CAI [14].

Both non-surgical and surgical treatments have been used to manage CAI. Approximately 80% of patients improve after undergoing non-surgical treatments through wearing orthosis, taping, brace on the ankle joint, and strength exercises for the peroneal muscle; however, 20% require surgical treatment [15,16,17]. Although various surgical methods are available for ankle ligament repair, such as the Chrisman–Snook and Elmslie procedures, the Modified Broström Operation (MBO) has gained popularity among clinicians and patients in recent years [18,19]. In the realm of MBO, continual evolution is marked by developing and refining various methods and strategies.

Recent advances in minimally invasive treatments have led to the development of several surgical options for managing CAI, offering faster recovery compared to traditional open surgery [20]. Less invasive operations are preferred over traditional open surgeries. Popular surgeries with minimally invasive techniques, such as arthroscopic surgeries, include anterior cruciate ligament restoration and rotator cuff surgery [21]. The MBO is widely used to treat lateral CAI [19,22]. The arthroscopic MBO (AMBO) is a surgical technique for preserving the fibular tendon and suturing the proximal portion of the inferior extensor retinaculum to the fibula, alleviating side effects of ankle joint stiffness [23] and preventing damage to the fibular tendon and sacral nerve owing to problems with tendon fixation [24]. Postoperatively, a rehabilitation exercise program is usually recommended for functional recovery of the ankle joint and the return to daily life and sports, with a focus on the reduction of ankle joint pain, recovery of the normal range of motion (ROM), increase in muscular strength and endurance, power, neuromuscular recovery, and improvement of balance, cardiorespiratory endurance, and functional ability [25].

Mangwani et al. [26] reported that inversion and eversion muscle training is necessary for ankle stabilization by controlling muscle strength recovery and equilibrium of ankle joint plantar flexion and dorsiflexion. Balance exercises for ankle stabilization and static and dynamic balancing exercises were used to activate the inversion, eversion, supination, and pronation muscles. Static balance exercise refers to the ability to maintain the body in an upright position in space against the force of gravity on a fixed base surface, while dynamic balance exercise refers to the ability to maintain a posture without falling over while the body is moving [27]. Accordingly, supination and pronation motions at the subtalar joint are crucial for maintaining foot stability [28]. However, exercise programs using bottom-up instability devices such as TOGU and BOSU balls are mainly used as ankle balance exercises [29]. 

This study aims to evaluate the impact of subtalar joint axis-based balance exercises on the thickness of the ATFL, ankle strength, and ankle stability after an AMBO for CAI. We hypothesized that balance exercises based on the subtalar joint axis after AMBO for patients with CAI would improve ankle strength, ATFL thickness, and ankle dynamic stability more than conventional balance exercises.

## 2. Materials and Methods

### 2.1. Participants

47 patients who underwent an AMBO for CAI were recruited from the Incheon Daechan Hospital (Republic of Korea). They were randomly divided into three groups, regardless of their affected sides: the control (CON, *n* = 11), general balance exercise (GBE, *n* = 17), and subtalar joint axis balance exercise (SBE, *n* = 19) groups. The sample size of this study was calculated using G-Power (ver. 3.1.9.7), with a significance level (α) of α = 0.05, power (1-β) of 0.80, and an effect size (*f*) of 0.48. With these parameters, the calculated sample size is 47 individuals. All patients were informed of the purpose and procedure of the study and signed a consent form before commencement. All participants in this study were diagnosed with ankle sprain grade III on MRI images prior to receiving AMBO treatment. After the AMBO, the affected side was immobilized with plaster and an air splint for 4 weeks, and the patients performed ROM recovery exercises for 2 weeks. Thereafter, all patients performed balance exercises for 6 weeks. Finally, ATFL thickness ultrasonography, dynamic ankle stability, and isokinetic muscle function were assessed 12 weeks after achieving a sufficient ROM. All study procedures were approved by the Research Ethics Committee at Sun Moon University (approval number: SM-202211-050-2). The physical characteristics of the participants are shown in Table 1.

### 2.2. Rehabilitation Program

Table 2 shows the rehabilitation program for the GBE group. The rehabilitation exercise program was modified from Brotzman and Wilke’s rehabilitation exercise program under the guidance of an exercise specialist [30]. Warm-up and cool-down were 10 min each, while the main exercise duration was 40 min, resulting in a total exercise time of 60 min, twice a week for 6 weeks. The rehabilitation exercise program comprised four phases. Phase 1 comprises ankle joint plantar flexion, dorsiflexion, eversion, and inversion exercises to prevent muscle atrophy caused by immobilization for 4 weeks postoperatively. Isometric exercises, isotonic exercises, partial weight bearing, and open kinetic chain exercises were performed within a range that allowed for gradual ROM improvement, with a focus on preventing pain.

In week 6 of phase 2, after achieving a normal ROM of the ankle joint, isotonic exercises, full weight bearing, and closed chain exercises were performed on both the contralateral and ipsilateral sides as strength training exercises. Each group performed proprioception and balance exercises, starting with a static balance exercise. 

In week 8 of phase 3, balance and functional exercises were performed. The former included static and dynamic balance exercises. In the static balance exercise, resistance was applied through dumbbell and barbell weights that were “somewhat hard” according to the patient’s motor perception of muscle ability. For the functional exercise, exercises such as squat, lunge, and side lunge were performed to strengthen the ankle and leg muscles.

In week 10 of phase 4, dumbbell and barbell weights were used for both static and dynamic balance, and functional exercises were performed. Jumping and running routines that may be employed in sports and daily life were used as functional exercises.

The exercises were performed on either the contralateral or ipsilateral side, and between sets, the ankle ROM, pain changes, and intensity adjustments were assessed. A 1 min rest period was provided between sets. Immediately after each exercise, an ice pack was applied to the affected area to prevent inflammation.

### 2.3. Physical Therapy for Control Group 

The control group’s physical therapy program comprised 3 min of cryotherapy and 20 min of transcutaneous electrical nerve stimulation, totaling 23 min for 1–3 weeks. Heat therapy was conducted for 10 min twice a week for 12 weeks, starting 4–6 weeks post-surgery. Cryotherapy was performed using a cryo-injection device, the Cryo-T 2 model (Metrum Cryoflex Sp. z o. o., Sp. k., Łomianki, Poland), which delivered cryogenic carbon dioxide liquid gas at −78 °C to the affected area. Subsequently, a transcutaneous electrical nerve stimulation device (ESPURGE, ITO Co., Ltd., Tokyo, Japan) was used to produce a pulsation frequency ranging between 2 and 10 Hz, adjusted to an intensity that the patient found comfortable [31].

### 2.4. GBE

The balance pad used for GBE (AIREX^®^ Balance Pad, Sins, Switzerland) is commonly employed in clinical practice for balance training on unstable surfaces. The patients started performing balance exercises 6 weeks postoperatively; static and dynamic balance exercises were performed for 6 weeks thereafter (i.e., 12 weeks postoperatively). Static balance was maintained by flexing the hip while standing on one foot, and three sets of 10 repetitions lasting 5 s each were performed using dumbbells and a barbell for progressive loading. Dynamic balance exercises focused on lower extremity strength and involved single squat and single Y-squat exercises. They were also performed for 5 s, with 3 sets of 10 repetitions using dumbbells and a barbell for progressive loading. Balance exercises were conducted for a duration of 15 min.

### 2.5. SBE

**Figure 1:**
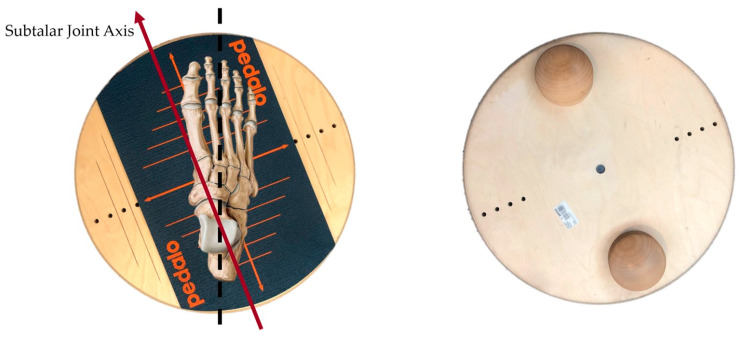
SBE applies balance movements to the foot in the direction of the subtalar joint axis using a circular pedalo to activate ankle eversion muscles, inversion muscles, lisfranc joint, and chopart joint movements (Pedalo^®^, Grasleben, Germany). SBE: subtalar joint axis balance exercise.

Subtalar joint axis balance exercises were conducted using a specialized exercise tool called Pedalo (Grasleben, Germany). This tool is designed to rotate in the sagittal plane of the foot, creating an angle of about 16 degrees with a circular motion of 50 cm in diameter, aligning with the subtalar joint axis. Participants placed their feet on the rotating Pedalo and engaged in balance exercises that focused on the muscles responsible for supination and pronation of the foot (Figure 1). The exercises commenced 6 weeks postoperatively and continued until 12 weeks postoperatively. Both static and dynamic balance exercises were performed. For static balance training, participants were required to maintain their balance by flexing the hip joint while standing on a single leg. They completed three sets of 10 repetitions, with each repetition lasting for 5 s. To progressively increase difficulty and resistance, participants incorporated dumbbells and barbells into the exercise. Dynamic balance exercises were aimed at enhancing lower limb strength and involved movements such as single squat and single Y-squat exercises. Again, participants performed three sets of 10 repetitions for each of these dynamic exercises, with a focus on gradually adding more weight by using dumbbells and barbells. The entire balance exercise routine had a duration of 15 min, and the exercise was carefully designed to enhance balance, strengthen the lower limbs, and facilitate the recovery process postoperatively. The unique Pedalo exercise tool allowed for a specialized approach to subtalar joint axis balance exercises.

### 2.6. Ultrasonography

The same examiner assessed ATFL thickness using musculoskeletal ultrasonography (MSUS). The ultrasound measurements were evaluated by a highly experienced orthopedic surgeon with over 15 years of experience to minimize measurement variability and increase reliability. All images from ultrasonography examinations were stored on a picture archiving and communication system (PACS). An ultrasound image of the ATFL was obtained using a 5–13 MHz multifrequency linear transducer (Model Logiq 6 compact ultrasound machine; General Electric Company, Waukesha, WI). Because the ATFL is a superficial structure, the examiner set the transducer at a frequency of 12 MHz and image depth of 2.5 cm to capture the clearest, highest resolution image possible. The examination was conducted on the part connecting the anterior border and lateral side of the talar neck [32]. Ultrasonic gel (Aquasonic 100; Parker Laboratories, Inc., Fair-Field, NJ, USA) was used to reduce friction with the skin, and the examination was performed within 10 min. Digitally obtained lateral ankle ultrasonography examination images were uploaded to PACS, and ATFL thickness was assessed.

### 2.7. Isokinetic Muscle Functions

Isokinetic muscle functions of the affected ankle were measured using the Biodex system 4 model (Biodex Medical Systems, Inc., Shirley, NY, USA). To measure inversion and eversion strength, participants performed three repetitions of each preliminary exercise before measuring ankle function. Ankle function was assessed with low-speed movements at 60°/s five times within a pain-free range of motion. The total exercise duration was 15 min. The ankle joint movement range was performed within a pain free range [33]. Exercise duration was 15 min.

### 2.8. Dynamic Stability

Dynamic stability was measured using a Y-Balance test (YBT) kit (Functional movement system, Danville, VA, USA). The proprioception and dynamic balancing ability of ankle joints was measured in three directions: anterior, posterior inner, and posterior outer [34]. The dynamic stability of the ankle joint was measured on both the affected and unaffected sides. After three practice sessions, the three aforementioned directions were measured from the center of the equipment in a single-leg stance three times for each lower extremity; the highest value was recorded.

If a participant (i) lost their balance, (ii) leaned on the upper section of the box to reach further, (iii) hit the box and lost contact with it, (iv) lost contact with the box during the pushing phase, or (v) failed to return the reaching foot to the starting position, the attempt was dismissed.

The composite scores were computed by summing the reach distances of the anterior, posteromedial, and posterolateral directions, dividing the total by three times the participant’s leg length, and then multiplying by 100 to obtain a percentage. The distance between the anterior superior iliac spine and the most distal region of the medial malleolus was measured [35,36]. The rest period between tests was 20 s, and the total testing duration was 15 min.

### 2.9. Statistical Analysis

For the data in this study, the mean and standard deviation were calculated using IBM SPSS 22.0 (SPSS Corp., Chicago, CA, USA), and the subtalar joint axis balance exercise effect was analyzed using a two-way repeated measures analysis of variance (ANOVA). Post hoc analysis was performed using the Tukey method, and the Bonferroni method was used to adjust the confidence interval. Statistical significance was set at α = 0.05.

## 3. Results

### 3.1. Change in Ultrasonography Results of ATFL Thickness

**Figure 2:**
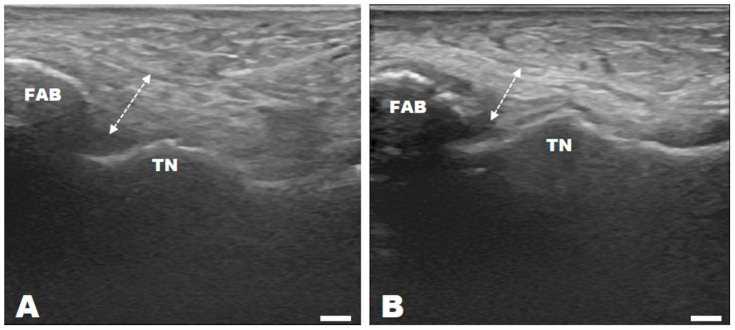
Ultrasonography for the extensor retinaculum and anterior talofibular ligament. The double dashed arrow is a musculoskeletal ultrasound image of the extensor retinaculum and anterior talofibular ligament thickness measurement (scale bar = 5 mm). (**A**) Ultrasound image after 6 weeks of AMBO, (**B**) ultrasound image after 12 weeks of AMBO and after balance exercise for 6 weeks. AMBO, arthroscopic modified Broström operation, FAB: fibula anterior border, TN: talus neck.

Table 3 and Figure 2 show the ATFL thickness obtained via ultrasonography. The two-way repeated measures ANOVA revealed that, while no significant difference in ATFL thickness was observed between groups, a significant interaction was observed between group and time [*F*(2, 44) = 16.556, *p* = 0.000], and a significant change was observed in thickness over time [*F*(1, 44) = 109.649, *p* = 0.000]. Ultrasonography examination of ATFL thickness did not reveal any significance in between-group differences before and after treatment. 6 weeks after treatment, no significant differences were observed between the CON and GBE groups. However, SBE demonstrated decreased ATFL thickness compared with the CON (*p* = 0.008) and GBE (*p* = 0.033) groups, achieving the normal range. Significant differences in pre- and post-treatment values were observed in all three groups: CON (*t* = 3.112, *p* = 0.011), GBE (*t* = 8.239, *p* = 0.000), and SBE (*t* = 8.877, *p* = 0.000).

### 3.2. Changes in Isokinetic Muscle Strength Power

Table 4 presents the changes in isokinetic muscle strength power. For ankle EV isokinetic muscle strength power, the two-way repeated measures ANOVA indicated a significant interaction between group and time [*F*(2,44) = 9.508, *p* = 0.000] and a significant effect of time [*F*(1,44) = 43.685, *p* = 0.000], but no significant differences were found between the groups. Ankle EV isokinetic muscle strength power did not reveal significant differences between groups before treatment. After 6 weeks of treatment, no significant difference between the CON and GBE groups was observed. However, the SBE group demonstrated a significant difference in the strength of the ankle EV muscle compared with the CON (*p* = 0.011) and GBE (*p* = 0.045) groups, confirming that subtalar joint axis balance exercise improves outcomes. The CON group did not demonstrate any significant differences in the pre- and post-treatment results 6 weeks postoperatively (*t* = −1.431, *p* = 0.183). Significant differences were found in the GBE (*t* = −3.579, *p* = 0.003) and SBE (*t* = −6.867, *p* = 0.000) groups.

Regarding ankle IV isokinetic muscle strength power, the two-way repeated measures ANOVA revealed a significant interaction between group and time [*F*(2,44) = 12.815, *p* = 0.000] and a significant effect of time [*F*(1, 44) = 62.930, *p* = 0.000], but no significant differences were observed between the groups. Ankle IV isokinetic muscle strength power did not show significant differences between groups before treatment. After 6 weeks of treatment, no significant differences between the CON and GBE groups were observed. However, in SBE, a significant difference was observed in the strength of the ankle IV muscle compared with the CON (*p* = 0.012) and GBE (*p* = 0.048) groups, confirming that the strength of the SBE group improved. The CON group did not demonstrate any significant differences in pre- and post-treatment results 6 weeks postoperatively (*t* = −1.882, *p* = 0.089). Significant differences were found in the GBE (*t* = −3.895, *p* = 0.001) and SBE (*t* = −8.652, *p* = 0.000) groups.

### 3.3. Changes in Dynamic Stability

Table 5 presents the changes in dynamic stability. The two-way repeated measures ANOVA indicated a significant interaction between group and time [*F*(2,44) = 5.762, *p* = 0.006] and a significant effect of time [*F*(1,44) = 59.831, *p* = 0.000], but no significant differences were found between groups. Dynamic stability did not exhibit significant differences between groups before treatment. After 6 weeks of treatment, no significant differences were observed between the CON and GBE groups. However, the SBE group demonstrated a significant difference in balance ability compared with the CON (*p* = 0.000) and GBE (*p* = 0.000) groups. The SBE group also demonstrated significantly improved ankle stability. All three groups demonstrated significant differences in the pre- and 6-week post-treatment results: CON (*t* = −2.573, *p* = 0.028), GBE (*t* = −5.922, *p* = 0.000), and SBE (*t* = −6.146, *p* = 0.000).

## 4. Discussion

This study aimed to investigate the changes in ATFL thickness and ankle strength and stability by comparing general balance and subtalar joint axis balance exercise methods for 6 weeks after AMBO in patients with CAI. Through this study, we aimed to validate the clinical efficacy of SBE and suggest a novel exercise approach for patients with CAI after undergoing AMBO.

ATFL primarily serves as the main stabilizer of the ankle joint and is the most vulnerable ligament in common lateral ankle sprain injuries [37]. Three phases in the ligament healing process occur immediately after surgical repair: inflammatory (7 days), proliferative (2–3 weeks), and maturation or remodeling (4–26 weeks) [38,39]. MSUS imaging is a novel technique used in the field of sports medicine [32]. Therefore, in this study, we used MSUS to assess ATFL thickness. Previous studies have shown that normal ATFL thickness is approximately 2 mm; however, partial tears increase it by more than 20%, and complete tears are not visible on imaging [40,41]. As both the extensor retinaculum and ATFL were sutured during the AMBO, the ATFL exhibited an increase in thickness to approximately 8 mm. ROM exercises begin after the proliferative phase, that is, during the ligament remodeling period, when Type III collagen is converted to the stronger Type I collagen, resulting in a decrease in the number of cells and density of blood vessels, as well as axial rearrangement of the collagen fibers [42].

In this study, after 6 weeks of exercise, ATFL thickness in the CON group decreased by 8.75%, while that of the GBE group decreased by 17.22%, but no significant difference was observed pre- and post-treatment. In contrast, in the SBE group, ATFL thickness decreased significantly by 33.93% (Pre: 8.99 ± 2.10 mm, Post: 5.94 ± 1.52 mm), approaching the normal range of thickness. Before exercise, there was no significant difference in ATFL thickness between groups. After 6 weeks of exercise, the SBE group exhibited the most substantial decrease in ATFL thickness among groups, with differences of 24.04% compared with the CON group and 18.18% compared with the GBE group.

In a previous study, patients with CAI showed morphological changes in the ATFL with the normal range as a reference, resulting in increased ATFL thickness and ankle instability [40]. In this study, ankle stability, manifested as the change in ATFL thickness, was reduced through postoperative exercise, with the SBE group demonstrating the greatest reduction.

Muscle function is crucial in CAI treatment [43]. CAI muscle strength training focuses on dorsiflexion strength to compensate for the lateral ankle instability caused by the initial ligament tear [44,45]. However, our study focused on the application of subtalar joint axis balance exercise. Clinically, subtalar joint movement is classified as inversion and eversion [46], and the ankle lateral muscles that act most in the motion of ankle inversion and eversion can be divided into the peroneal muscle and tibialis posterior muscle. The peroneal muscle is weaker than the tibialis posterior muscle [47]. It plays an important role in ankle inversion and prevents hyper inversion [47]. It also protects the ligament and joint upon ankle inversion, and its strength and functional recovery lead to improved control during ankle eversion and inversion [48,49].

Isokinetic muscle function is categorized into strength and endurance, which are effective in measuring a muscle’s maximal exercise capacity [50]. Peak torque is considered the gold standard for estimating skeletal strength [51].

In this study, after 6 weeks of exercise, the CON group’s EV muscle strength (60°/s) increased by 11.57%, while that of the GBE increased by 21.22%, but no significant pre- and post-treatment differences were observed. In contrast, the SBE group showed the greatest change in EV muscle strength (60°/s) at 48.61% (pre: 9.99 ± 5.59 N.M; post: 19.44 ± 4.22 N.M). Before exercise, there was no significant difference in EV muscle strength (60°/s) between groups. After 6 weeks of exercise, the SBE group demonstrated the most substantial increase in EV muscle strength (60°/s), with differences of 28.4% compared with the CON group and 19.55% compared with the GBE group.

Additionally, the IV muscle strength (60°/s) of the CON group increased by 24.54%, while that of the GBE group increased by 25.76%; however, no significant differences were observed. In contrast, the SBE group showed the greatest change in IV muscle strength (60°/s) at 56.39% (pre: 10.69 ± 7.89 N.M; post: 24.51 ± 9.29 N.M). Before exercise, there was no significant difference in IV muscle strength (60°/s) between groups. After 6 weeks of exercise, the SBE group demonstrated the most substantial increase in IV muscle strength (60°/s), with differences of 36.15% compared with the CON group and 24.77% compared with the GBE group.

Muscle weakness is associated with CAI, and strength training is an essential part of rehabilitation to prevent instability [52]. Decreased ankle strength contributes to balance loss, and enhanced ankle muscle strength has been shown to improve balance recovery [53,54].

Static and dynamic postural stability deficits have been reported to be the most important factors in the treatment of patients with CAI [55,56]. Weakness in the muscles and ligaments of the ankle can decrease static and dynamic balance abilities, resulting in instability of the center of gravity during various movements, including walking, changing direction, and landing [57]. Previous studies have shown reduced dynamic stability in the affected ankle of patients with CAI [56,58]. Additionally, CAI leads to subtalar joint instability, affecting overall pronation balance and supination forces operating across the subtalar joint axis by stabilizing the foot joints during weightbearing activities [59]. YBT is widely used to assess patients with CAI [60]. Many studies demonstrated the effects of various exercises on the symptoms of acute ankle sprains in CAI [61]. Typical rehabilitation exercises for ankle sprains comprise ROM exercises, isometric and isotonic strength training, and proprioceptive exercises [62]. Balance board exercises have been reported to improve ankle strength and balance [63], and coordination exercises such as the one step exercise have been reported to improve ankle strength and proprioception [64]. Generally, patients with CAI are often prescribed general balance exercises. However, the application of exercise for subtalar joint axis balance is yet to be well examined.

In this study, after 6 weeks of exercise, the dynamic stability of the CON group slightly increased by 5.51%, while that of the GBE group increased by 12.84%, but no significant pre- and post-treatment differences were observed. In contrast, the SBE group’s dynamic stability increased significantly by 17.57% (pre: 79.92 ± 11.35%; post: 96.95 ± 4.52%), indicating that this exercise was good for ankle stability. Before exercise, there was no significant difference in dynamic stability among groups. After 6 weeks of exercise, the SBE group demonstrated the most substantial increase in dynamic stability, with differences of 10.11% compared with the CON group and 7.92% compared with the GBE group.

These findings were similar to those of Anguish and Anguish [65], who reported improvements in balance ability after a balance training program for patients with CAI. Therefore, as evidenced by this study, SBE decreased the change in ATFL thickness after AMBO, increasing ankle ROM and improving pain due to the increased strength of EV and IV, ultimately enhancing ankle stability.

Moreover, the uniqueness of the balance exercise in this study is that it was performed on a larger area of the sole of the foot during the GBE. However, in the balance exercise of the SBE, the circular Pedalo was used in the direction of the subtalar joint axis to reduce the area of the sole through which ground reaction force is transmitted, possibly resulting in improvements in the ankle ROM.

This study had several limitations. The number of patients participating in the 6-week study was limited, and when assessing only the treatment effect, a longer period of follow-up is needed to confirm long-term effects and continuous improvement. Because some evaluation tools, such as ultrasonography, which was used to measure ATFL thickness, may have limits, the use of multiple evaluation tools is recommended. The height of the Pedalo used in the SBE is 5 cm, meaning that the height of the tool needs to be improved by lowering it while keeping stability in mind. Moreover, research into the development of exercise tools is also necessary. These limitations necessitate a more careful evaluation of the study results, and further studies addressing these limitations are warranted.

## 5. Conclusions

This study showed that subtalar joint axis balance exercise decreased ATFL thickness and increased muscle strength and dynamic stability after AMBO. Based on the present results, subtalar joint axis balance exercise can be used to improve ankle stability and is necessary in research and clinical trials for various conditions. Future studies with extended term applications and exercise tools that are both suitable and secure for subtalar joint axis balance exercise are warranted.

## Figures and Tables

**Table 1:** Baseline data of the study participants.

	Sex		Involved		Age (Years)	Height (cm)	Weight (kg)	BMI (kg/m^2^)
	M	F	Rt	Lt				
CON (*n* = 11)	5	6	3	8	34 ± 14.76	169.55 ± 9.05	76.55 ± 21.76	26.34 ± 5.97
GBE (*n* = 17)	8	9	5	12	28.53 ± 10.93	171 ± 10.20	67.82 ± 16.67	23.01 ± 4.44
SBE (*n* = 19)	5	14	9	10	28.53 ± 9.75	165.16 ± 8.62	62.79 ± 12.78	22.83 ± 3

The data are presented as the mean ± standard deviation of the mean. BMI: body mass index, CON: control, GBE: general balance exercise, SBE: subtalar joint axis balance exercise.

**Table 2:** Rehabilitation exercise program.

	Index	Exercise	Intensity	Side
Warm up	Flexibility	Begin gentle ROM exercises of the ankle (DF, PF, IV, EV)	4–6 weeks (5 s × 10 reps) × 3 sets	Involved
Intensive muscle exercise	Isometric	Ankle towel isometric exercise (DF, PF, IV, EV)	4–5 weeks (5 s × 10 reps) × 3 sets	Involved
	Isotonic	Towel pulling with toe Ankle tubing exercise (DF, PF, IV, EV)	5–6 weeks (5 s × 10 reps) × 3 sets	Involved
		Toe raise Calf raise	5–6 weeks (5 s × 10 reps) × 3 sets	Both
	Functional	Squat Lunge Side lunge Single step up (box)	6–12 weeks (5 s × 10 reps) × 3 sets	Both
		Jump (jump: pain free jump in place) Hop (hop: one leg-jump hop)	6–12 weeks 10 reps × 3 sets	
		Running	10–12 weeks 30 min	
	Static balance	Single standing HF Single standing HF and SF (DB, BB)	6–12 weeks (5 s × 10 reps) × 3 sets	Both
	Dynamic balance	Single squat (DB, BB) Single Y-squat (DB, BB)	8–12 weeks 10reps × 3 sets	
Cool down	Stretching	Foam roller	10 min	Both

ROM: range of motion, DF: dorsi flexion, PF: plantar flexion, IV: inversion, EV: eversion, HF: hip flexion, SF: shoulder flexion, DB: dumbbell, BB: barbell.

**Table 3:** Changes in ATFL thickness obtained via ultrasonography. Units: mm.

		Pre	Post	Paired *t*-Test		Two-Way RM ANOVA		
				*t*-Value	*p*-Value		*F*	*p*
ATFL thickness via ultrasonography	CON (*n* = 11)	8.57 ± 1.99 ^a^	7.82 ± 2.11 ^a^	3.112	0.011 *	G	0.692	0.506
	GBE (*n* = 17)	8.77 ± 1.88 ^a^	7.26 ± 1.88 ^a^	8.239	0.000 ***	T	109.649	0.000 ***
	SBE (*n* = 19)	8.99 ± 2.10 ^a^	5.94 ± 1.52 ^b^	8.877	0.000 ***	G×T	16.556	0.000 ***

Data are presented as the mean ± standard deviation of the mean. Two-way RM ANOVA: two-way repeated measures analysis of variance. mm: millimeter, CON: control, GBE: general balance exercise, SBE: subtalar joint axis balance exercise, G: group, T: time, G×T: group × time. Values marked with different lowercase letters indicate a statistically significant difference between groups (*p* < 0.05). Significant differences in pre- and post-test values (*p* < 0.05). * *p* < 0.05, *** *p* < 0.001.

**Table 4:** Changes in isokinetic muscle strength power before and after rehabilitation exercises (60°/s). Units: N.M.

		Pre	Post	Paired *t*-Test		Two-Way RM ANOVA		
				*t*-Value	*p*-Value		*F*	*p*
EV	CON (*n* = 11)	12.31 ± 4.76 ^a^	13.92 ± 5.11 ^a^	−1.431	0.183	G	0.445	0.644
	GBE (*n* = 17)	11.54 ± 5.75 ^a^	15.64 ± 6.87 ^a^	−3.579	0.003 **	T	43.685	0.000 ***
	SBE (*n* = 19)	9.99 ± 5.59 ^a^	19.44 ± 4.22 ^b^	−6.867	0.000 ***	G×T	9.508	0.000 ***
IV	CON (*n* = 11)	11.81 ± 6.27 ^a^	15.65 ± 7.26 ^a^	−1.882	0.089	G	0.873	0.425
	GBE (*n* = 17)	13.69 ± 8.34 ^a^	18.44 ± 9.43 ^a^	−3.895	0.001 **	T	62.930	0.000 ***
	SBE (*n* = 19)	10.69 ± 7.89 ^a^	24.51 ± 9.29 ^b^	−8.652	0.000 ***	G×T	12.815	0.000 ***

Data are presented as the mean ± standard deviation of the mean. Two-way RM ANOVA: two-way repeated measures analysis of variance. N.M: newton meter, EV: eversion, IV: inversion, CON: control, GBE: general balance exercise, SBE: subtalar joint axis balance exercise, G: group, T: time, G×T: group × time. Values marked with different lowercase letters indicate a statistically significant difference between groups (*p* < 0.05). Significant differences in pre- and post-test values (*p* < 0.05). ** *p* < 0.01, *** *p* < 0.001.

**Table 5:** Changes in dynamic stability. Units: %.

		Pre	Post	Paired *t*-Test		Two-Way RM ANOVA		
				*t*-Value	*p*-Value		*F*	*p*
Dynamic stability	CON (*n* = 11)	82.35 ± 6.9 ^a^	87.15 ± 7.43 ^a^	−2.573	0.028 *	G	2.624	0.084
	GBE (*n* = 17)	77.79 ± 10.32 ^a^	89.25 ± 5.59 ^a^	−5.922	0.000 ***	T	59.831	0.000 ***
	SBE (*n* = 19)	79.92 ± 11.35 ^a^	96.95 ± 4.52 ^b^	−6.146	0.000 ***	G×T	5.762	0.006 **

Data are presented as the mean ± standard deviation of the mean. Two-way RM ANOVA: two-way repeated measures analysis of variance. %: percent, CON: control, GBE: general balance exercise, SBE: subtalar joint axis balance exercise, G: group, T: time, G×T: group × time. Values marked with different lowercase letters indicate a statistically significant difference between groups (*p* < 0.05). Significant differences in pre- and post-test values (*p* < 0.05). * *p* < 0.05, ** *p* < 0.01, *** *p* < 0.001.

## Data Availability

Data are contained within the article.
